# IDPEnsembleTools: An open‐source library for analysis of conformational ensembles of disordered proteins

**DOI:** 10.1002/pro.70427

**Published:** 2025-12-23

**Authors:** Hamidreza Ghafouri, Giacomo Janson, Silvio C. E. Tosatto, Alexander Miguel Monzon

**Affiliations:** ^1^ Department of Biomedical Sciences University of Padova Padova Italy; ^2^ Department of Biochemistry and Molecular Biology Michigan State University East Lansing Michigan USA; ^3^ Institute of Biomembranes, Bioenergetics and Molecular Biotechnologies National Research Council (CNR‐IBIOM) Bari Italy

**Keywords:** conformational ensembles, dimensionality reduction, intrinsically disordered proteins, Protein Ensemble Database (PED), structural ensemble analysis

## Abstract

Intrinsically disordered proteins (IDPs) lack stable tertiary structure and instead exist as dynamic ensembles of conformations, playing essential roles in cellular regulation, signaling, and disease. As structural ensembles of IDPs become increasingly available through databases such as the Protein Ensemble Database (PED) and various computational generation methods, the need for systematic tools to analyze and compare these ensembles has grown. Here, we present IDPET (Intrinsically Disordered Protein Ensemble Tools), an open‐source Python library designed to facilitate comprehensive analysis of IDP conformational ensembles. IDPET enables users to load and process ensembles from various sources and formats in parallel, compute global and local structural features, perform dimensionality reduction and clustering, and compare ensembles quantitatively using metrics based on Jensen–Shannon divergence (JSD). To demonstrate the package's functionalities, we analyze three ensembles of the unfolded drkN SH3 domain deposited in PED. This example illustrates how IDPET can extract structural descriptors, visualize conformational diversity, assess global and local features, and quantify differences between ensembles generated using distinct experimental and computational methods. By providing a reproducible and extensible framework, IDPET supports systematic exploration of ensemble features in IDPs. It is compatible with atomistic and coarse‐grained models and can be easily integrated with community resources.

## 1 INTRODUCTION

Intrinsically disordered proteins and regions (collectively referred to as IDPs) play vital roles in diverse biological and pathological processes across both prokaryotic and eukaryotic organisms. Unlike folded proteins, IDPs lack stable tertiary structures under physiological conditions, allowing them to participate in dynamic cellular functions such as signal transduction, genome maintenance, cell division, circadian rhythm regulation, and homeostasis (Cuylen et al. 2016; Dunker et al. 2001; Pelham et al. 2020; Wright and Dyson 2015).

Due to their dynamic nature, IDPs are best represented by conformational ensembles, which capture their structural variability. These ensembles consist of rapidly interconverting states, often assigned statistical weights to reflect their relative significance (Bonomi et al. 2017). From these ensembles, various global and local descriptors such as radius of gyration (*R*_g_), transient secondary structure content, Flory scaling exponent, solvent‐accessible surface area (SASA), and end‐to‐end distances (*R*_ee_) can be extracted (Holehouse and Kragelund 2023). These properties capture how sequence composition and contextual factors shape the conformational behavior of IDPs (Martin et al. 2020).

Importantly, ensemble properties can reveal evolutionary and functional patterns that are not readily apparent from primary sequences alone. For instance, Gonzalez‐Foutel et al. (González‐Foutel et al. 2022) demonstrated that the mean end‐to‐end distance is conserved across orthologs of a specific disordered linker, despite limited sequence similarity. Thus, while traditional multiple sequence alignments (MSAs) often fail to detect conservation in IDPs (Ruff and Pappu 2021) ensemble‐level features offer a promising alternative for exploring sequence–ensemble–function relationships. This, in turn, depends on generating accurate conformational ensembles.

This typically requires the integration of experimental data, including nuclear magnetic resonance (NMR) (Gibbs et al. 2017), small‐angle X‐ray and neutron scattering (SAXS and SANS) (Kikhney and Svergun 2015), single‐molecule Förster resonance energy transfer (sm‐FRET) (Schuler et al. 2016), and cryogenic electron microscopy (Cryo‐EM) (Bonomi and Vendruscolo 2019), with computational techniques such as atomistic simulations, coarse‐grained models, Monte Carlo methods, and knowledge‐based approaches (Joseph et al. 2021; Piana et al. 2020; Tesei et al. 2024; Vitalis and Pappu 2009). More recently, machine learning (ML) models and data‐driven force fields have enabled ensemble generation directly from sequence data (Janson et al. 2023; Tesei and Lindorff‐Larsen 2023), even in the absence of explicit experimental inputs.

However, this process is inherently ambiguous: most experimental measurements reflect average behaviors rather than unique conformations. Consequently, multiple distinct ensembles may be consistent with the same data (Ravera et al. 2016). A well‐known example is the Sic1 protein, which has over 10 distinct ensembles archived in the Protein Ensemble Database (PED) (Ghafouri et al. 2024), each generated using different experimental or computational protocols.

To systematically analyze and compare such ensembles, we developed IDPET, “Intrinsically Disordered Protein Ensemble Tool,” a Python package designed for structural and statistical analysis of IDP ensembles. IDPET enables users to load multiple ensembles simultaneously, compute ensemble‐level properties, and visualize key features through interactive plots. The tool also includes modules for dimensionality reduction, clustering, and ensemble comparison and can retrieve relevant datasets through integrated database APIs. These capabilities allow users to quantify differences between ensembles generated by different methods or conditions. In the following sections, we describe the structure and functionality of IDPET and demonstrate its application using an example IDP from the PED.

## 2 RESULTS

This section describes the general workflow and the various analyses modules in IDEPT. We use the package to analyze three IDP ensembles of the SH3 domain of drkN protein, highlighting its core features. To further demonstrate the versatility of IDPET, additional analyses were performed on systems with diverse structural and experimental characteristics. These include (i) multidomain protein ensembles containing both folded and intrinsically disordered regions and (ii) five α‐synuclein ensembles encompassing both atomistic and coarse‐grained representations. These examples, available as Jupyter Notebooks at https://github.com/BioComputingUP/EnsembleTools/tree/main/notebooks, confirm that IDPET efficiently handles a wide range of protein architectures, sizes, and ensemble types.

### 2.1 IDPET workflow

The IDPET package is organized into three main modules: *ensembl*e, *ensemble_analysi*s, and *visualization*. The *ensemble* module is responsible for loading structural ensembles into the package. IDPET uses MDTraj (McGibbon et al. 2015) for parsing coordinate data, allowing it to support various formats, including .xtc, .dcd, and .pdb. Users can also specify a particular chain or region of interest by providing the chain ID and residue indices. Currently, only one chain per ensemble can be analyzed at a time. Only protein atoms are loaded in IDPET (e.g., solvent is excluded). Both atomistic and coarse‐grained (Cα‐only) protein ensembles are supported. User‐provided trajectory files are assumed to be free of discontinuities caused by periodic boundary conditions. If unit‐cell information is available, users can let IDPET reconstruct molecules that are split across periodic boundaries via MDTraj's built‐in functionalities.

**FIGURE 1:**
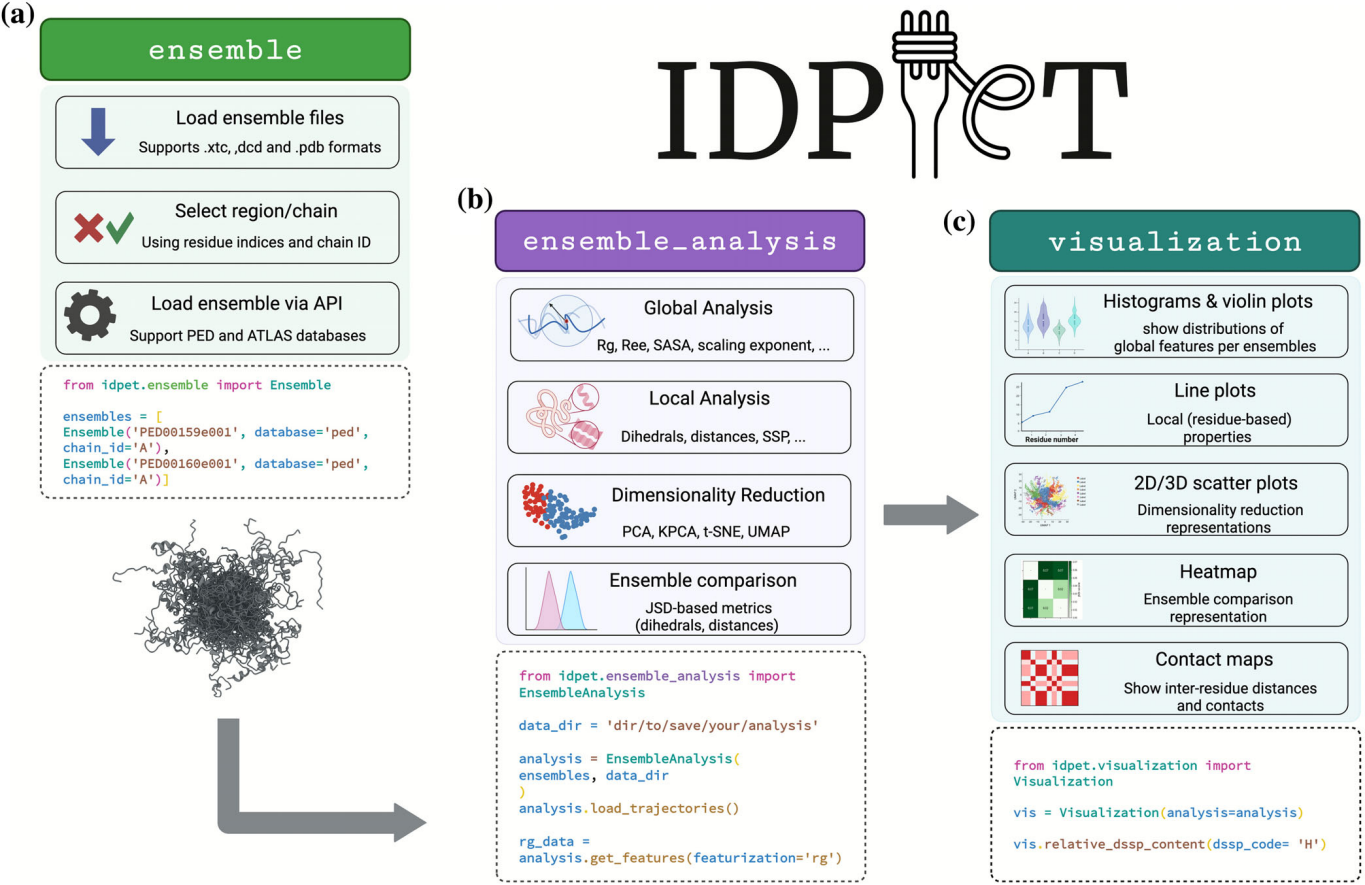
Overview of IDPET architecture and analysis workflow. (a) The *ensemble* module handles input of structural ensembles from local files (.xtc, .dcd, .pdb) or directly via API from PED and ATLAS. Users can specify chain and residue selections for targeted analysis. (b) The *ensemble_analysis* module extracts structural features at global (e.g., *R*_g_, scaling exponent) and local levels (e.g., backbone dihedrals, distances, secondary structure propensity). It supports dimensionality reduction (PCA, KPCA, t‐SNE, UMAP) and ensemble comparison using Jensen–Shannon divergence metrics. (c) The *visualization module* generates interactive plots including global feature distributions (e.g., histograms, violin plots), residue‐level properties (e.g., line plots), dimensionality reduction projections (2D/3D scatter plots), contact maps, and ensemble similarity heatmaps. Example Python code snippets illustrate how each module can be used.

Three dimensional coordinate data can be imported either from local files or directly from external databases. Currently, IDPET supports API requests from PED and ATLAS (Vander Meersche et al. 2024), allowing users to retrieve ensembles using their entry IDs in these databases (Figure 1a).

Once the ensemble is loaded, the *ensemble_analysis* module performs the core analysis through four main pipelines (Figure 1b):

- Global analysis: Extracts overall structural features, such as *R*_g_, *R*_ee_, asphericity, prolateness, global solvent‐accessible surface area (SASA), and the Flory scaling exponent (Alston et al. 2023).
- Local analysis: Computes residue‐level properties, including intramolecular distances, dihedral angles, and site‐specific order and flexibility parameters that reflect local disorder (Jeschke 2024).
- Dimensionality reduction: Implements three algorithms, t‐SNE, UMAP, and PCA, to project high‐dimensional structural data (e.g., distances, angles, root mean square deviation) into 2D or 3D space. This helps compare ensembles and visualize differences in internal geometry. Additionally, conformations can be clustered using the k‐nearest neighbors (KNN) algorithm (Appadurai et al. 2023).
- Ensemble comparison: Introduces three comparison scores based on Jensen–Shannon divergence (JSD), which quantify differences between distributions of structural features (e.g., dihedral angles, distances) across ensembles. These scores are presented as comparison matrices.

Following this stage, data are organized either as 1D arrays (for global features, yielding one value per conformation) or as 2D arrays (for residue‐level descriptors, providing one value per residue per conformation), enabling downstream analysis and visualization.

The third module, *visualization* (Figure 1c), handles graphical representation of the analysis results. All outputs from the *ensemble_analysis* module can be visualized and saved through this interface. For global features, IDPET provides histograms and violin plots. Distance‐based data can be visualized using contact probability maps, with customizable thresholds and color schemes. Local features like site‐specific order, flexibility, or secondary structure content are shown as line plots, where the x‐axis corresponds to residue number and the y‐axis to the property value. For dimensionality reduction, IDPET generates scatter plots in 2D or 3D space. Points can be colored using various labels, such as ensemble identity, clustering results, or global features like *R*_g_, *R*_ee_, asphericity, prolateness, and SASA.

### 2.2 Studying the global and local features of conformational ensembles of drKN SH3 domain

The drkN SH3 domain, derived from the Drosophila Downstream of receptor kinase (Drk) adaptor protein, is a well‐characterized model system in protein folding and disorder studies. In its folded state, it adopts a canonical SH3 β‐barrel structure, while its unfolded state serves as a prototypical intrinsically disordered protein (IDP). This duality makes it an ideal benchmark for assessing conformational ensemble modeling techniques. In this work, we analyze three ensembles of the unfolded drkN SH3 domain deposited in the Protein Ensemble Database (PED): PED00156e001, PED00157e001, and PED00158e001. These ensembles were generated and characterized by Lincoff et al. (Lincoff et al. 2020), each using different conformer generation strategies followed by refinement through the Extended Experimental Inferential Structure Determination (X‐EISD) approach (Lincoff et al. 2020).

The ensemble PED00156e001 was constructed by selecting 100 structures from a large pool of ~100,000 conformers generated using TraDES (Feldman and Hogue 2002), a tool that systematically unfolds the native structure without applying experimental restraints. This “random” ensemble represents a broad sampling of disordered conformational space, ranging from compact to highly extended structures. PED00157e001, by contrast, consists of 100 conformers drawn from a smaller pool (*n* = 1700) biased toward fitting SAXS and NMR chemical shift data, using the ENSEMBLE (Krzeminski et al. 2013) approach to guide structure selection. The third ensemble, PED00158e001, includes 88 conformations derived from a 50:50 mixture of the TraDES and ENSEMBLE pools, refined against the same experimental data. This hybrid strategy combines broad conformational diversity with experimentally informed structure selection.

To demonstrate the capabilities of IDPET in capturing ensemble‐level diversity, we initially conducted a two‐part analysis focusing on both global and local structural features.

#### 2.2.1 Global analysis

**FIGURE 2:**
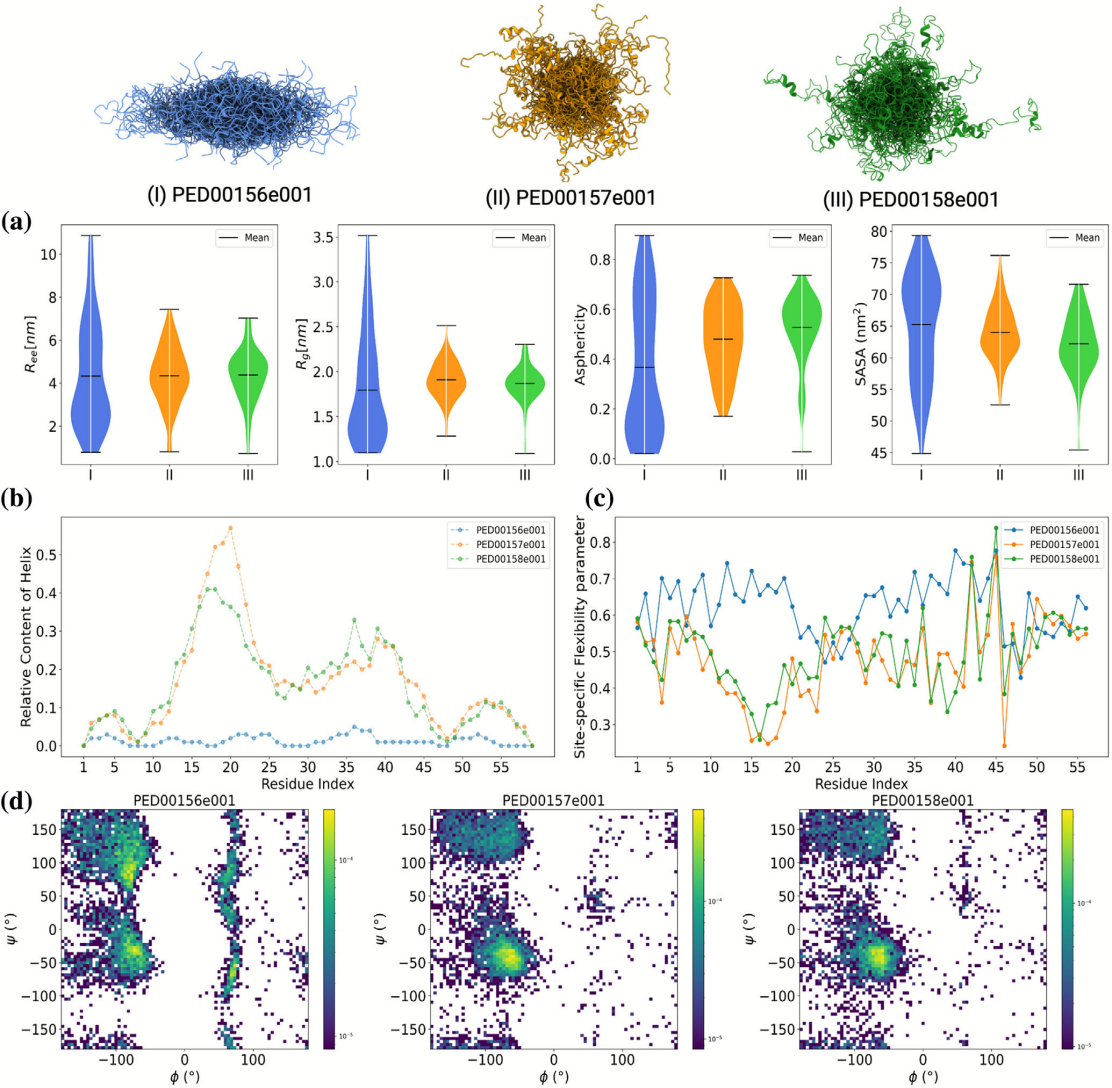
Analyzing the global and local features of the ensembles of the drkN SH3 domain in the unfolded state deposited in PED. Cartoon representations (top) illustrate the conformational ensembles PED00156e001 (I, blue), PED00157e001 (II, orange), and PED00158e001 (III, green), with colors corresponding to those used in the plots. (a) Global conformational properties analyzed with IDPET, showing distributions of the (*R*_g_), R_ee_, asphericity, and SASA. While the three ensembles exhibit roughly similar mean values for each descriptor, their distributions differ, reflecting distinct structural sampling. Notably, PED00156e001 was generated from a randomly generated pool by progressive unfolding of the native structure, while PED00157e001 and PED00158e001 were derived from experimentally biased pools. (b) Relative content of Alpha Helix per residue, highlighting higher local helicity in PED00157e001 and PED00158e001. (c) Site‐specific flexibility profiles indicate increased conformational variability for PED00156e001, particularly in regions spanning residues 15–20 and 30–45. (d) Ramachandran plots (2D histograms of backbone dihedral angles ϕ and ψ) reveal enrichment of α‐helical conformations in PED00157e001 and PED00158e001, in contrast to the broader conformational spread observed in PED00156e001.

We first examined global descriptors such as *R*_g_, *R*_ee_, asphericity, and SASA. These parameters offer a macroscopic view of each ensemble's structural landscape. As shown in Figure 2a, the three ensembles exhibit roughly similar ensemble‐averaged values for most global features; however, their distributions differ markedly. The ensemble PED00156e001 shows broader and more heterogeneous distributions across all global metrics, reflecting its derivation from a large and unconstrained conformational pool. This suggests a greater diversity in compactness and shape, with populations spanning collapsed, intermediate, and extended states. In contrast, PED00157e001 and PED00158e001, both biased by SAXS and NMR data, exhibit narrower distributions centered around more compact, helical‐like conformations. These ensembles display reduced standard deviations and lower asphericity, indicative of more homogeneous structural populations.

#### 2.2.2 Local analysis

Next, we investigated local conformational features at the residue level. Using IDPET, we computed the per‐residue helical content for each ensemble, revealing distinct regions of transient secondary structure. PED00157e001 and PED00158e001 display prominent α‐helical propensities in sequence regions 16–20, 30–45, and 50–55 (Figure 2b). These features are absent in PED00156e001, which retains the disordered coil‐like characteristics typical of a purely statistical ensemble. To further probe local heterogeneity, we calculated site‐specific flexibility indices based on the circular variance of backbone φ and ψ dihedral angles. The regions with increased helicity in PED00157e001 and PED00158e001 also show reduced flexibility (Figure 2c), suggesting the presence of partial ordering induced by the NMR chemical shift restraints used during refinement.

We complemented these analyses with Ramachandran plots to visualize the dihedral angle distributions (Figure 2d). While PED00156e001 shows a broad spread of conformations typical of disordered regions, PED00157e001 and PED00158e001 exhibit distinct enrichment in the α‐helical regions of φ/ψ space. These observations confirm that the application of experimental restraints during ensemble generation has a significant effect on local conformational preferences, even if global averages remain similar.

In summary, our global and local analyses of the drkN SH3 domain ensembles highlight the impact of initial conformer pools and experimental data integration on the structural features of refined ensembles. IDPET enables systematic comparison of such differences and provides both macroscopic and microscopic insights into IDP ensemble characteristics.

### 2.3 Mapping the conformational space of drkN SH3 domain ensembles via t‐SNE

To explore how different ensemble generation strategies influence the conformational diversity of the unfolded drkN SH3 domain, we applied dimensionality reduction using *t*‐distributed stochastic neighbor embedding (t‐SNE) as implemented in IDPET. This technique allows high‐dimensional structural data to be projected into a two‐dimensional space (Appadurai et al. 2023), offering an interpretable view of the ensemble landscape and facilitating comparisons across datasets.

For each ensemble (PED00156e001, PED00157e001, PED00158e001), we constructed a feature matrix from three types of structural descriptors: (i) pairwise root mean square deviation (RMSD) between conformers, (ii) backbone φ and ψ dihedral angles, and (iii) inter‐residue Cα–Cα distances. These features provide complementary perspectives on structural variability, capturing both global geometric differences and local conformational states.

**FIGURE 3:**
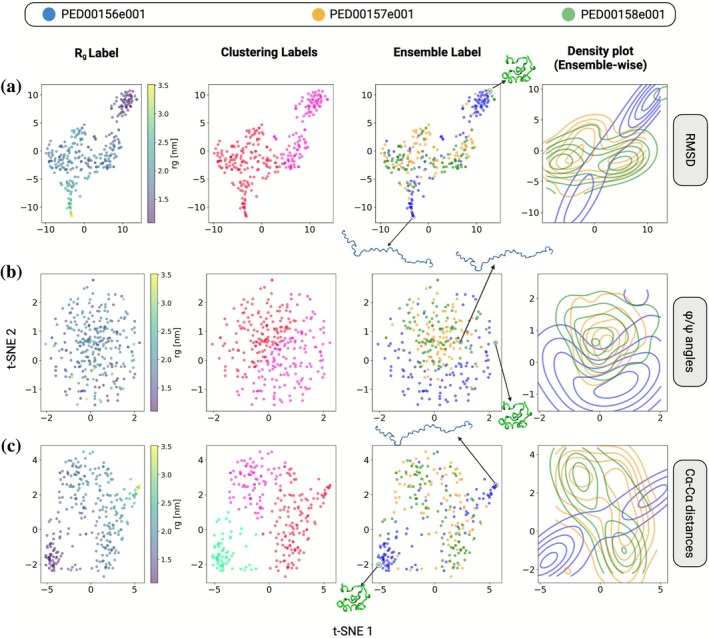
Visualization of drkN SH3 conformational ensembles in reduced‐dimensional space using t‐SNE projections based on different structural descriptors. Each row corresponds to a different input feature used to compute pairwise distances between conformers: (a) Cα–Cα distances, (b) RMSD, and (c) backbone dihedral angles (ϕ, ψ). These features were used to generate 2D t‐SNE embeddings for three PED ensembles (PED00156e001, PED00157e001, PED00158e001), shown as columns from left to right: (1) conformers colored by radius of gyration (*R*_g_), (2) K‐nearest neighbor clustering labels, (3) original ensemble labels, and (4) kernel density estimates (KDEs) for each ensemble in t‐SNE space. Panels in the third column include the same two representative structures, corresponding to a compact and an extended conformation from PED00158e001and PED00156e001, respectively, overlaid across all rows to illustrate how these structural extremes are positioned within the t‐SNE embeddings.

When using RMSD‐based features, each structure is represented as a vector of its distances to all others in the ensemble. The resulting t‐SNE projection (Figure 3a) reveals that PED00157e001 and PED00158e001 occupy compact and overlapping regions in the 2D embedding, consistent with their narrower global feature distributions. In contrast, PED00156e001 spans a wider region, reflecting its broader structural heterogeneity. This observation aligns with earlier analyses, in which this ensemble showed greater variance in radius of gyration and asphericity.

To determine whether these structural embeddings could be used to discriminate between ensembles, we applied KNN clustering to the t‐SNE output. Although the RMSD‐based embedding broadly separated structures by their compactness, it did not fully resolve the three ensembles, suggesting that RMSD alone lacks the resolution to capture finer structural differences.

We therefore repeated the dimensionality reduction using torsional features, specifically, the backbone φ and ψ dihedral angles, as input (Figure 3b). This representation more effectively distinguished the ensembles. PED00156e001 adopts a distinct distribution, while PED00157e001 and PED00158e001, both refined using similar experimental restraints, show substantial overlap. The improved resolution highlights the discriminatory power of local angular features, which capture subtle conformational biases introduced by ensemble refinement protocols.

Finally, we assessed a third feature set based on Cα–Cα inter‐residue distances. The resulting t‐SNE projection resembled the RMSD‐based embedding in its global structure (Figure 3c), capturing differences in ensemble compactness but offering limited discrimination at finer levels. Clustering on this embedding produced three partially overlapping groups, which correlated well with global structural descriptors such as radius of gyration and end‐to‐end distance.

Taken together, these dimensionality reduction results underscore the importance of feature selection in comparative ensemble analysis. While global distance‐based descriptors are useful for capturing broad structural trends, local features such as torsional angles offer enhanced sensitivity to protocol‐specific biases. IDPET provides flexible access to both types of representations and facilitates systematic evaluation of conformational space across ensembles.

### 2.4 Quantitative ensemble comparison using Jensen–Shannon divergence

To enable systematic comparison between conformational ensembles of disordered proteins, IDPET implements three complementary metrics based on Jensen–Shannon divergence (JSD) (Lin 2002) (Ensemble comparison section in Data S1). These scores assess the dissimilarity between feature distributions extracted from different ensembles, capturing both global and local structural differences. The three implemented metrics are:

- adaJSD (alpha distance average JSD): compares the distributions of all pairwise Cα–Cα distances, reflecting global structural differences.
- ramaJSD (Ramachandran JSD): evaluates the joint φ/ψ backbone dihedral angle distributions, sensitive to local backbone geometry. This metric applies only to all‐atom ensembles.
- ataJSD (alpha torsion average JSD): quantifies differences in α‐torsion angles formed by four consecutive Cα atoms, offering a coarse‐grained measure of local structure that is compatible with both all‐atom and coarse‐grained ensembles.

**FIGURE 4:**
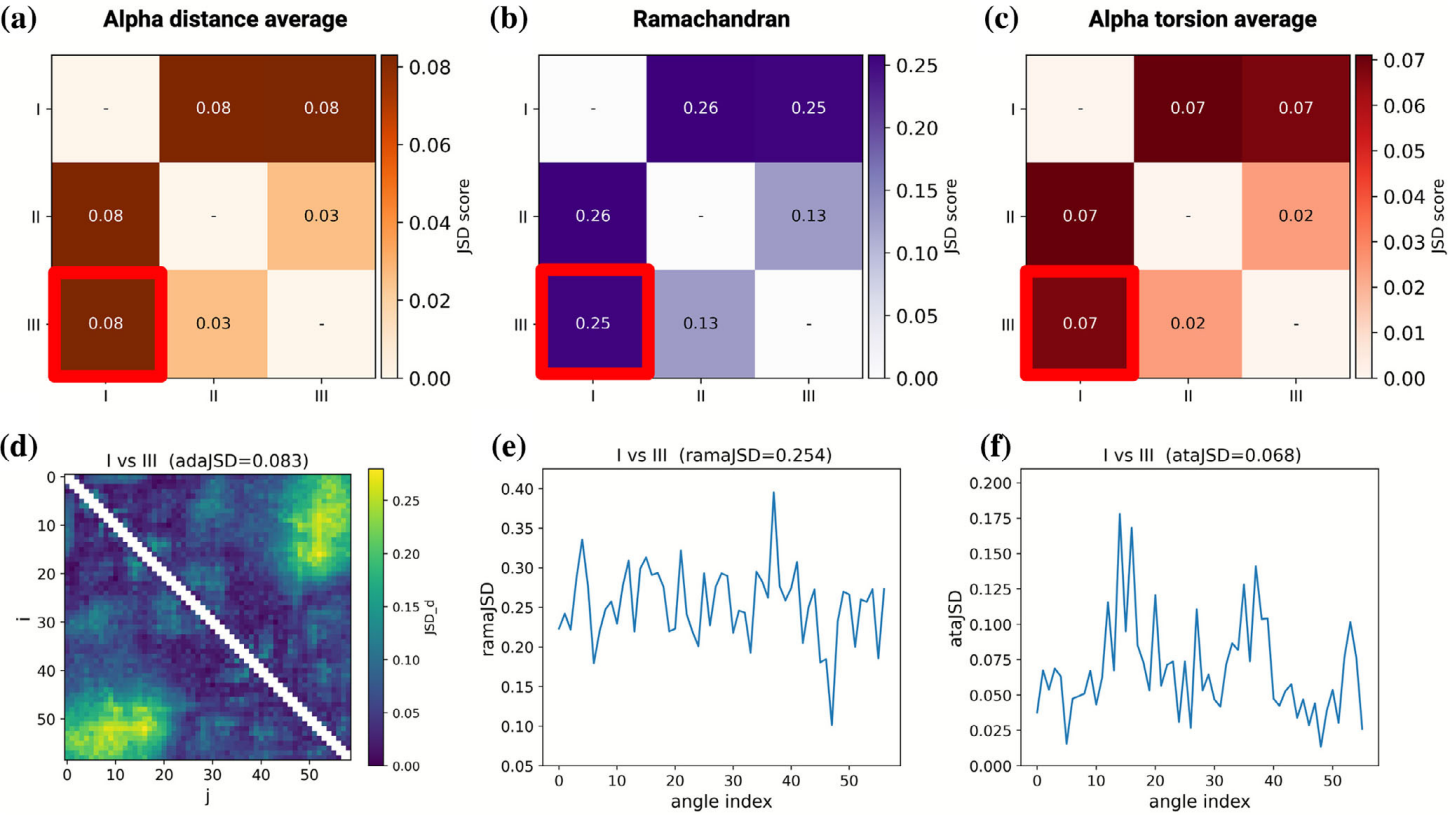
Quantitative comparison of drkN SH3 ensembles using Jensen–Shannon divergence (JSD) across structural features. Pairwise comparison between three PED ensembles (I: PED00156e001, II: PED00157e001, III: PED00158e001) using Jensen–Shannon divergence (JSD) to assess differences in structural descriptors. Top row: heatmaps showing average pairwise JSD scores between ensembles based on (a) Cα–Cα distances, (b) backbone dihedral (φ/ψ) angles, and (c) α‐torsion angles. Bottom row: residue‐level or feature‐wise JSD profiles for the most divergent pair (I vs. III, highlighted in red). Panels show (d) a heatmap of per‐residue JSD scores for Cα–Cα distances, (e) residue‐wise JSD for dihedral angles, and (f) residue‐wise JSD for α‐torsion angles.

We applied these metrics to compare the three ensembles of the drkN SH3 domain: PED00156e001(I), PED00157e001(II), and PED00158e001(III). As shown in Figure 4, each metric provides a distinct perspective on ensemble similarity.

The adaJSD values (Figure 4a) reveal that ensembles II and III are the most similar in terms of global Cα–Cα distance distributions, consistent with the result of dimensionality reduction analysis. In contrast, ensemble I shows higher divergence when compared to both II and III, reflecting its broader conformational variability. This suggests that while the overall shape and compactness remain in a comparable range, ensemble I explores a more diverse set of global conformations.

By contrast, the ramaJSD scores (Figure 4b) show greater divergence, particularly in comparisons involving ensemble I. This indicates that local backbone dihedral angle distributions differ substantially between the randomly sampled ensemble and those refined using experimental restraints. These results align with our earlier observations that ensemble II and III exhibit enriched helical propensities, while ensemble I explores a broader range of disordered conformations.

The ataJSD metric (Figure 4c) reveals a similar trend to ramaJSD, though with lower absolute values. This behavior reflects the coarser resolution of α‐torsion angles compared to φ/ψ angles, yet still confirms meaningful local structural divergence, especially in distinguishing PED00156e001 from the other two ensembles. Importantly, ataJSD offers robustness against noise in atomistic detail, making it a useful alternative when working with lower‐resolution or coarse‐grained models.

To assess the statistical significance of these differences, we implemented a bootstrapping strategy within IDPET. For each pair of ensembles, the metric is recalculated over multiple iterations using subsampled conformers, and the distributions are compared via a one‐sided Mann–Whitney *U* test. This allows for estimation of intra‐ versus inter‐ensemble divergence, even for ensembles with small sample sizes (Figures S1 and S2 and Bootstrap section in Data S1).

Finally, while panels a–c in Figure 4 show average JSD scores that summarize global differences between ensembles, panels d–f provide a more detailed, residue‐level view. These plots show how Cα–Cα distances (adaJSD), Ramachandran angles (ramaJSD), and α‐torsions (ataJSD) vary across the sequence when comparing ensemble I with ensemble III, as highlighted by the red boxes in Figure 4a–c. The largest differences appear in the N‐terminal (residues 10–20) and C‐terminal (residues 45–58) regions, which correspond to segments with transient helices in ensemble III. This suggests that using experimental restraints can lead to more structured regions in specific parts of the sequence. In contrast, the middle region is relatively consistent between the two ensembles. Together, these local profiles help explain where and how ensembles differ, beyond what global metrics can capture.

Overall, this ensemble comparison framework demonstrates that while global properties may converge across ensembles, local structural features, particularly backbone dihedral distributions, are more sensitive to the underlying modeling strategy. IDPET thus provides a multiresolution toolkit for ensemble comparison, capable of capturing subtle yet functionally relevant differences in IDP conformational landscapes.

## 3 DISCUSSION

The highly dynamic nature of IDPs is best captured by structural ensembles that represent the diversity of their conformational states. Traditionally, such ensembles have been derived from molecular dynamics (MD) simulations, often combined with ensemble refinement techniques to integrate experimental data (e.g., NMR, SAXS, smFRET) (Bottaro and Lindorff‐Larsen 2018). Alternatively, knowledge‐based methods such as TraDES (Feldman and Hogue 2002) and Flexible‐Meccano (Ozenne et al. 2012) generate large pools of random conformers, which can then be reweighted to match experimental observations (Bernadó et al. 2007). More recently, machine learning and data‐driven approaches have made it possible to generate conformational ensembles even in the absence of direct experimental input. For example, idpGAN (Janson et al. 2023) uses a generative framework to produce realistic ensembles directly from sequence data. Furthermore, new methods have emerged that generate ensembles based on AlphaFold2 (Jumper et al. 2021) predictions (Brotzakis et al. 2025; Schnapka et al. 2025; von Bülow et al. 2025a). For instance, AFflecto (Pajkos et al. 2025) utilizes AlphaFold predictions for the globular regions of proteins while sampling conformations for the disordered segments, such as loops, linkers, and terminal tails. Alternatively, data‐driven force fields like CALVADOS (Tesei et al. 2021; von Bülow et al. 2025b) which are optimized using SAXS and NMR measurements of IDPs, enable the generation of realistic coarse‐grained ensembles.

These recent advances have significantly expanded both experimentally derived and computationally predicted structural ensembles. For example, the number of entries in the PED has grown from 87 in 2021 to over 500 today. In parallel, large‐scale efforts have also led to the publication of predicted conformational ensembles for the entire human intrinsically disordered proteome (Tesei et al. 2024).

This increasing diversity and volume of ensembles now demand analytical frameworks that are not only flexible but also tailored to the unique challenges of IDPs, including low‐resolution representations and high conformational heterogeneity.

While general‐purpose libraries like MDTraj (McGibbon et al. 2015) and MDAnalysis (Michaud‐Agrawal et al. 2011) offer extensive functionality for MD trajectory analysis, they are not tailored to the particularities of IDP ensemble evaluation. This has led to the development of more specialized tools. SOURSOP (Lalmansingh et al. 2023), for instance, provides IDP‐specific analysis pipelines built on top of MDTraj, supporting tasks such as secondary structure assignment, contact analysis, and the calculation of various global and local properties from MD trajectories of IDP simulations. MMMx (Jeschke 2024) focuses on integrative ensemble modeling guided by DEER spectroscopy, particularly well‐suited for spin‐labeling studies. EnGens (Conev et al. 2023) facilitates generation, featurization, and dimensionality reduction of ensembles, streamlining the analysis of large‐scale conformational datasets. A detailed comparison between IDPET and SOURSOP is provided in Table S2.

In this context, IDPET introduces a complementary and modular toolkit designed for the structural ensemble analysis of disordered proteins. Its key features include support for simultaneous multi‐ensemble analysis, a comprehensive set of global and local structural descriptors, and flexible pipelines for dimensionality reduction and visualization. These capabilities make IDPET particularly well‐suited for comparing ensembles generated using different computational or experimental methods. In addition to single‐domain IDP ensembles, IDPET also supports multidomain proteins and disordered regions within folded proteins, which can be loaded by specifying residue ranges or chain selections. This enables targeted analyses of flexible linkers and domain–domain interactions using the same set of structural descriptors. Notably, it integrates JSD‐based metrics and bootstrapping procedures to enable statistically robust comparisons of ensemble similarity, even in the presence of noise or limited sample sizes. While the drkN SH3 example serves as a validation case, the same analysis pipelines can be applied to functionally or evolutionarily related IDPs to assess how ensemble properties (e.g., compaction, flexibility, transient structure) correlate with conserved biological roles, regulatory mechanisms, or environmental conditions.

Despite these advances, important challenges remain. The accuracy and interpretability of ensemble‐derived features are deeply influenced by the underlying model quality and the choice of descriptors. Ensemble properties can vary depending on the simulation method, refinement strategy, and biophysical context, complicating direct comparisons (Ghafouri et al. 2025). Feature selection for dimensionality reduction and similarity analysis is particularly nontrivial. Linear methods like PCA may miss important nonlinear relationships, while nonlinear techniques like t‐SNE and UMAP are sensitive to initialization and hyperparameter tuning. Moreover, one‐ or two‐dimensional similarity metrics such as JSD may overlook higher‐order correlations between structural features, an issue partly addressed by more computationally intensive approaches like WASCO (González‐Delgado et al. 2023). IDPET addresses some of these limitations by supporting multiple structural representations—such as RMSD matrices, backbone torsions, and inter‐residue distances—and by offering modular analysis pipelines adaptable to both atomistic and coarse‐grained data. Its bootstrapping‐based statistical evaluation framework further enhances the robustness of ensemble comparisons. Future extensions of IDPET will include integration of sequence‐based descriptors to connect sequence features (e.g., charge patterning, hydropathy, and aromatic content) with ensemble‐level structural properties. This will further expand IDPET into a unified framework for correlating sequence composition with conformational behavior in disordered and hybrid proteins.

## 4 CONCLUSION

In summary, IDPET provides an easy‐to‐use and comprehensive toolbox for studying structural ensembles of IDPs. The modular characteristics of the package also give this possibility to continuously update the package based on the coming state‐of‐the‐art methods corresponding to featurization, comparison, multidimensional projection, and new visualization settings. Moreover, the package can be deployed as an analysis pipeline for developing standalone web application interfaces. Future developments will include the integration of IDPET analysis pipelines within the PED, enabling automatic computation and visualization of ensemble‐level descriptors for all deposited entries. This integration will strengthen the connection between data deposition and analysis, facilitating large‐scale comparative studies across disordered protein ensembles.

## 5 METHODS

IDPET was developed as a modular Python library for analyzing conformational ensembles of intrinsically disordered proteins. Its architecture comprises three main modules: *ensemble* for loading data, *ensemble_analysis* for extracting structural features, and *visualization* for plotting outputs. Ensemble properties are computed using a combination of MDTraj‐based and custom algorithms. Global features include radius of gyration, end‐to‐end distance, and solvent‐accessible surface area; local features include dihedral angles, flexibility indices, and residue‐level order parameters.

Dimensionality reduction is performed using PCA, Kernel PCA, UMAP, and t‐SNE on diverse structural representations (e.g., φ/ψ angles, α‐torsions, pairwise RMSD). To assess ensemble similarity, we implemented three Jensen–Shannon divergence (JSD)‐based scores: adaJSD (global distances), ataJSD (α‐torsions), and ramaJSD (backbone dihedrals). Statistical significance of comparisons can be evaluated through a bootstrapping procedure with Mann–Whitney *U* testing. All figures were generated using matplotlib and Plotly, with interactive options available. Additional implementation details, feature definitions, dimensionality reduction strategies, and ensemble comparison formulas are provided in detail in Methods section in Data S1.

IDPET's computational efficiency scales with its NumPy, MDTraj, and scikit‐learn dependencies. On standard workstations, typical analyses such as ensemble loading, featurization, computation of global descriptors, dimensionality reduction via PCA, and JSD‐based ensemble comparisons complete within seconds to minutes for ensembles comprising up to several thousand conformers. More computationally intensive tasks, including SASA calculations and t‐SNE projections, may require longer runtimes that increase nonlinearly with ensemble size. Benchmarks performed on large PED ensembles (~3000 conformers) are provided in Table S1 and detailed in the online documentation.

Comprehensive documentation for the IDPET package (https://biocomputingup.github.io/EnsembleTools/) was developed using Sphinx, ensuring accessibility and clarity. A dedicated demo section was included to illustrate the package's capabilities in analyzing conformational ensembles of disordered proteins. Several Jupyter notebooks are also provided in the GitHub repository (https://github.com/BioComputingUP/EnsembleTools/tree/main/notebooks), offering examples of how to extract ensemble properties for diverse systems, including all‐atom, coarse‐grained, and multidomain proteins. In addition, the notebooks provide step‐by‐step guidance on loading ensembles into IDPET, comparing ensembles, and applying dimensionality‐reduction techniques to highlight structural differences.

## AUTHOR CONTRIBUTIONS

**Hamidreza Ghafouri:** Conceptualization; investigation; methodology; validation; visualization; writing – original draft; writing – review and editing; software; formal analysis; data curation. **Giacomo Janson:** Conceptualization; investigation; writing – original draft; methodology; validation; visualization; writing – review and editing; software; formal analysis; data curation. **Silvio C. E. Tosatto:** Supervision; resources; project administration; writing – original draft; writing – review and editing; conceptualization; funding acquisition. **Alexander Miguel Monzon:** Writing – original draft; writing – review and editing; project administration; formal analysis; supervision; conceptualization; methodology.

## FUNDING INFORMATION

This work has been supported by ELIXIR, the research infrastructure for life‐science data; COST Action ML4NGP [CA21160], supported by COST (European Cooperation in Science and Technology); European Union through NextGenerationEU; PNRR project ELIXIRxNextGenIT [IR0000010]; National Center for Gene Therapy and Drugs based on RNA Technology [CN00000041]; co‐funded by the European Union under grant agreement no. 101182949 (HORIZON‐MSCA‐SE project IDPfun2); views and opinions expressed are however those of the author(s) only and do not necessarily reflect those of the European Union or the European Research Executive Agency. Neither the European Union nor the granting authority can be held responsible for them. Funding for open access charge: University of Padua.

## CONFLICT OF INTEREST STATEMENT

The authors declare no conflicts of interest.

## Supporting information


**Data S1.** Supporting Information text.
**Table S1.** Description of the features that IDPET could extract from the ensembles.
**Figure S1.** Relationship between IDPET comparison scores and ensemble size.
**Figure S2.** It shows the result of bootstrapping by providing the confidence intervals.
**Table S1.** Wall‐clock times for common IDPET operations.
**Table S2.** Comparison between the IDPET and SOURSOP packages for analyzing conformational ensembles of IDPs.

## Data Availability

The data that support the findings of this study are openly available in GitHub at https://github.com/BioComputingUP/EnsembleTools.
