# Supplementary material for: IDPEnsembleTools: An open‐source library for analysis of conformational ensembles of disordered proteins
Source: Protein Sci. 2025 Dec 23;35(1):e70427. doi: 10.1002/pro.70427 (PMC12724005; doi:10.1002/pro.70427)
Supplement: Supplementary file 1 — Data S1. Supporting Information text. Table S1. Description of the features that IDPET could extract from the ensembles. Figure S1. Relationship between IDPET comparison scores and ensemble size. Figure S2. It shows the result of bootstrapping by providing the confidence intervals. Table S1. Wall‐clock times for common IDPET operations. Table S2. Comparison between the IDPET and SOURSOP packages for analyzing conformational ensembles of IDPs. [file PRO-35-e70427-s001.pdf]

IDPEnsembleTools: an open-source library for analysis of conformational ensembles of disordered proteins

Hamidreza Ghafouri[1,^], Giacomo Janson[2,^], Silvio C.E. Tosatto[1,3,*], Alexander Miguel Monzon[1,*]

[1]Department of Biomedical Sciences, University of Padova, Padova 35121, Italy

[2]Department of Biochemistry and Molecular Biology, Michigan State University, East Lansing, Michigan 48824, United States

[3]Institute of Biomembranes, Bioenergetics and Molecular Biotechnologies, National Research Council (CNR-IBIOM), Bari 70126, Italy

^ These authors contributed equally to this work.

*Corresponding authors. Department of Biomedical Sciences, University of Padova, Padova 35121, Italy.

E-mails: alexander.monzon@unipd.it (A.M.M.) and silvio.tosatto@unipd.it (S.C.E.T.)

Supplementary Material

Methods

Dimensionality reduction

IDPET offers PCA (Principal Component Analysis) and Kernel PCA (KPCA), t-SNE (t-distributed Stochastic Neighbor Embedding) and UMAP (Uniform Manifold Approximation and Projection) as three dimensionality reduction methods for studying the IDPs/IDRs ensembles' properties. These methods are applicable on extracted feature matrices from ensembles. Currently, IDPET can transform ensembles into six different feature matrices based on phi-psi angles, intra atomic Cɑ distances, pairwise RMSD, trRosetta-style phi angles, trRosetta-style omega angles and alpha angles (**Supplementary Table.1**). Then, based on the selected feature, each conformation in the ensemble will be transformed to a 1D array containing extracted feature data and consequently the ensembles of conformations will be introduced as a 2D matrix to the selected dimensionality reduction method.

Supplementary Table 1: Description of the features that IDPET could extract from the ensembles and proper dimensionality reduction techniques

Feature	Description	Dimensionality reduction methods
phi-psi angles	Phi and Psi are the dihedral angles around the N-Cɑ and Cɑ-C atoms respectively	KPCA, circular UMAP and t-SNE(using circular metric)
intra atomic Cɑ distances	Interatomic distances between Cɑ atoms	PCA, UMAP and t-SNE (using euclidean metric)
pairwise RMSD	Generate all-vs-all matrix contains all pairwise RMSD values between the conformations in the ensembles	UMAP and t-SNE (using precomputed metric for symmetric matrices)
trRosetta-style phi angles	The planar angle between three atoms of two residues i and j (Cɑ[i], Cb[i] and Cb[j])	KPCA, UMAP and t-SNE (using circular metric)
trRosetta-style omega angles	The dihedral angle between the planes formed by four atoms of 2 residues i and j (Ca[i], Cb[i], Cb[j], and Ca[j])	KPCA, UMAP and t-SNE (using circular metric)
alpha angles	The dihedral angle between the planes formed by four Cɑ atoms of 4 consecutive residues (Ca[i], Cɑ[i+1], Cɑ[i+2], and Cɑ[i+3])	KPCA, UMAP and t-SNE (using circular metric)

a. PCA & KPCA

IDPET implements user-friendly interfaces for PCA and KPCA using the scikit-learn library to explore conformational ensemble properties. KPCA is particularly suited for analyzing non-linear and periodic data such as angular variables. To account for the circular nature of angular features, each angle is transformed into its sine and cosine components before computing the kernel matrix using a custom distance-based kernel function. This transformation preserves angular periodicity and enables meaningful dimensionality reduction in the latent space, offering insight into conformational diversity.

b. t-SNE

As part of its dimensionality reduction toolkit, IDPET includes an adapted implementation of t-SNE based on the pipeline suggested by Robustelli et al. [1], which combines t-SNE with k-means clustering to effectively group heterogeneous conformational ensembles. Two hyperparameters are tuned: the number of k-means clusters and the t-SNE perplexity value, which balances local and global relationships within the data by approximating the number of nearest neighbors. IDPET performs a grid search over these parameters, selecting the optimal combination using the Silhouette score, a metric that evaluates both intra-cluster cohesion and inter-cluster separation to assess clustering quality.

c. UMAP

IDPET also includes UMAP, a non-linear dimensionality reduction technique based on fuzzy topological representations of data. UMAP generally outperforms linear methods in preserving complex structural relationships and is computationally more efficient than t-SNE, with comparable embedding quality. The two main hyperparameters are: the number of neighbors (analogous to t-SNE's perplexity), which governs the balance between local and global data structure, and the minimum distance between points, which controls cluster compactness versus preservation of global structure. IDPET adopts the same optimization strategy as for t-SNE, tuning these parameters by maximizing the Silhouette score across combinations of neighbor counts and cluster numbers.

Ensemble comparison

IDPET implements a set of scores based on Jensen–Shannon divergence (JSD) to quantitatively compare conformational ensembles of proteins with the same length LLL, even when their sequences differ. These scores evaluate the similarity between probability distributions of structural features, such as interatomic distances or torsion angles, derived from the ensembles. To compute the JSD for continuous variables, the values are first discretized into histograms with N_{bins}, following established practices [2]. Given two

distributions P and Q corresponding to a specific molecular feature from each ensemble, their JSD is defined as:

$$JSD(P \| Q) = \frac{1}{2}(KLD(P \| M) + KLD(Q \| M))$$

where $M = \frac{1}{2}(P + Q)$ is a mixture, and KLD is the Kullback-Leibler divergence, expressed as:

$$KLD(X \| M) = \sum_{k}^{N_{bins}} X_k log\left(\frac{X_k}{M_k}\right)$$

where k is the bin index and X_k and M_k are the frequencies for bin k estimated from histogram data, with $M_k = \frac{1}{2}\left(P_k + Q_k\right)$. No pseudo-counts are used and bins with $X_k = 0$ have zero contribution. JSD scores range from 0 (P and Q have identical counts) to a maximum of $\log log\ (2) \approx 0.6931$ (no bin has at least one count from both P and Q).

IDPET implements the following JSD based scores:

a. Carbon Alpha Distance Average JSD (adaJSD)

The adaJSD score compares Cα-Cα distances in two ensembles and is defined as:

$$adaJSD = \frac{1}{N_{pairs}} \sum_{j-i>1} JSD\left(D_{ij}^{[A]} \ \| \ D_{ij}^{[B]}\right)$$

where $D_{ij}^{[A]}$ and $D_{ij}^{[B]}$ are the distance distributions between residue i and j in ensembles A and B, and $N_{pairs} = (L - 1)(L - 2)/2$ is the total number of distances evaluated. For each distance, its histogram range is defined by its minimum and maximum values in the ensembles.

b. Alpha Torsion Average JSD (ataJSD)

The ataJSD score compares α angle distributions:

$$ataJSD = \frac{1}{N_{alpha}} \sum_{i=2} JSD\left(T_{i-1,i,i+1,i+2}^{[A]} \ \| \ T_{i-1,i,i+1,i+2}^{[B]}\right)$$

where $N_{alpha} = L - 3$ is the number α angles in a protein and $T_{i-1,i,i+2,i+3}^{[A]}$and $T_{i-1,i,i+2,i+3}^{[B]}$ are the distributions of α angles formed by residue i and its neighbors. The histogram range is always - π to π.

c. RAMAchandran plot average JSD (ramaJSD)

The ramaJSD (RAMAchandran plot average JSD) score compares joint φ and ψ angle distributions:

$$ramaJSD = \frac{1}{N_{rama}} \sum_{i=2} JSD\left(R_i^{[A]} \parallel R_i^{[B]}\right)$$

where $N_{rama} = L - 2$ is the number of residues with both φ and ψ values and $R_i^{[A]}$and $R_i^{[B]}$ are the joint distributions of φ and ψ for residue i. For both angles, the histogram range is always - π to π split into N_{bins}, resulting in a 2d histogram with an effective bin number of N_{bins}^2.

Effect of ensemble size

A crucial parameter for computing above mentioned scores is the number of bins N_{bins}. Large N_{bins} values will lead to inaccurate results if ensembles contain few conformers. While users can manually set N_{bins}, by default IDPET automatically assigns it via the square root rule:

$$N_{bins} = min(\lceil\sqrt{n}\rceil, 50)$$

where n is the number of conformers in the smaller ensembles. Our benchmarking (see **Supplementary Figure 1**) shows that IDPET scores reliably capture ensemble differences with as few as 25 conformers. However, when comparing smaller ensembles, the discriminative power of the scores significantly decreases.

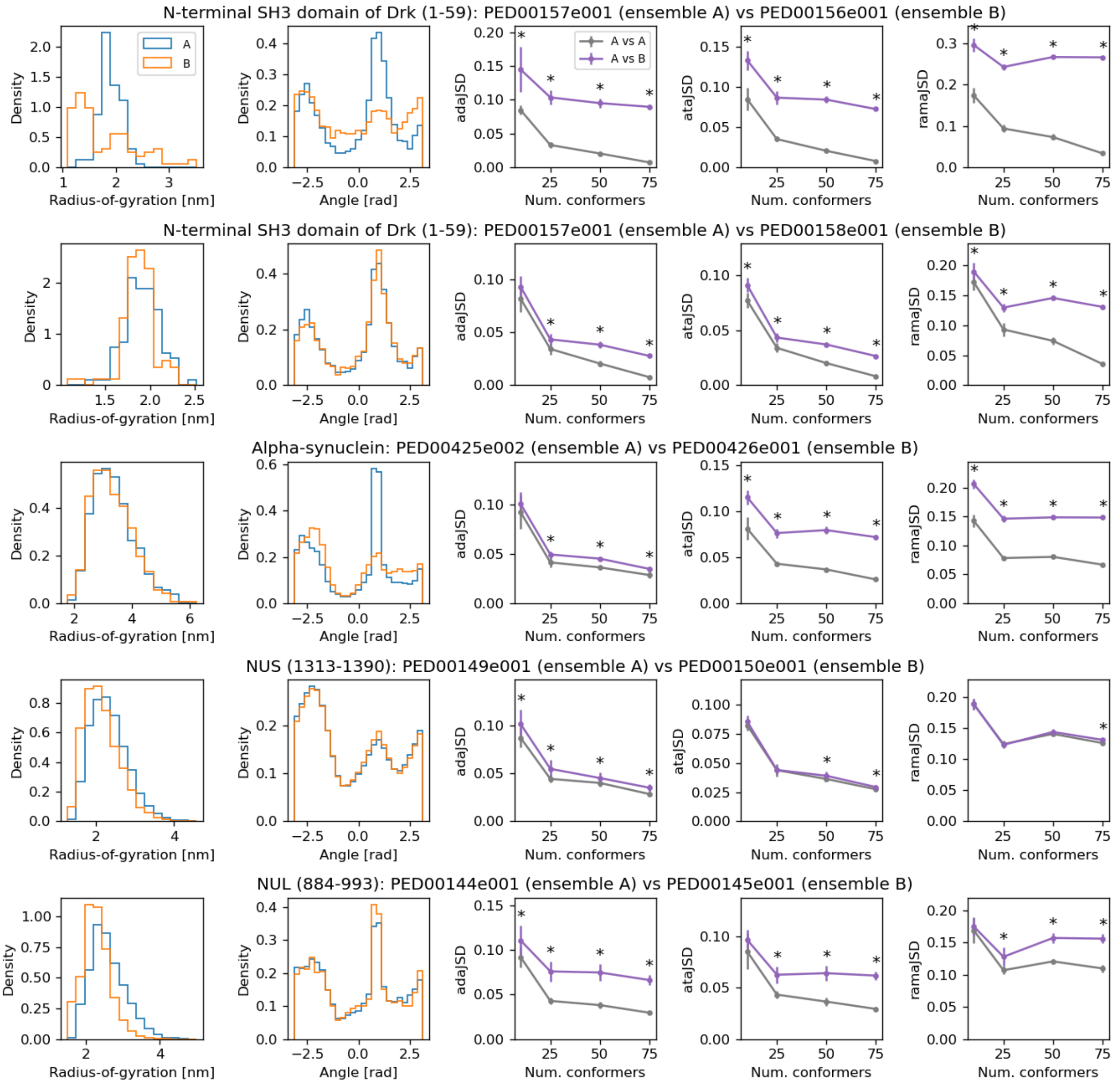

Supplementary Figure 1. Relationship between IDPET comparison scores and ensemble size. Each row corresponds to two PED ensembles (A and B) for the same protein. The first two columns show histograms of radius of gyration and α angles, summarizing global and local structural properties, respectively. The remaining columns plot adaJSD, ataJSD, and ramaJSD scores as a function of the number of bootstrapped conformers. Gray curves show A vs A auto-comparisons; purple curves show A vs B comparisons. Each point represents the average of n = 10 bootstrap replicates (error bars: standard deviation). Asterisks indicate statistically significant differences (Mann–Whitney U test, $p < 0.05$) between A vs A and A vs B scores for ensemble sizes of 10, 25, 50, and 75 conformers. When A and B share similar structural properties (first two columns), differences in JSD scores become indistinguishable from background variation at low ensemble sizes. These results highlight the need for caution when interpreting scores from small ensembles (fewer than 50 conformers).

Statistical analysis

Due to the finite size of protein ensembles analyzed in IDPET, a non-zero JSD score does not necessarily imply a statistically significant difference between two ensembles. To address this, IDPET includes an optional bootstrap-based statistical analysis, particularly recommended for small ensembles (e.g., fewer than 50 conformers).

This analysis can be applied to all comparison scores implemented in IDPET (adaJSD, ataJSD, and ramaJSD), and proceeds as follows:

1. Inter-ensemble bootstrapping:
   Conformers are randomly sampled (with replacement) from ensembles A and B and compared over $N_{bootstrap}$ iterations to generate a distribution of inter-ensemble scores.
2. Intra-ensemble bootstrapping:
   Each ensemble (A and B) is also bootstrapped and compared to itself $N_{bootstrap}$ times. The highest average intra-ensemble score from these self-comparisons is used as the reference.
3. Significance testing:
   A one-sided Mann–Whitney U test is applied to compare the distributions of inter-ensemble and intra-ensemble scores. The null hypothesis is that both distributions are equal; the alternative hypothesis is that the inter-ensemble scores are stochastically greater. The resulting p-value is reported to assess statistical significance.

Additionally, IDPET reports bootstrap confidence intervals for the score differences using the percentile method (**Supplementary Figure 2**). This approach allows users to gauge whether differences between ensembles are meaningful or arise from statistical noise. While this analysis is critical for small ensembles, it is generally unnecessary for large ensembles (e.g., >1,000 conformers), where comparison scores are already reliable estimators of structural divergence.

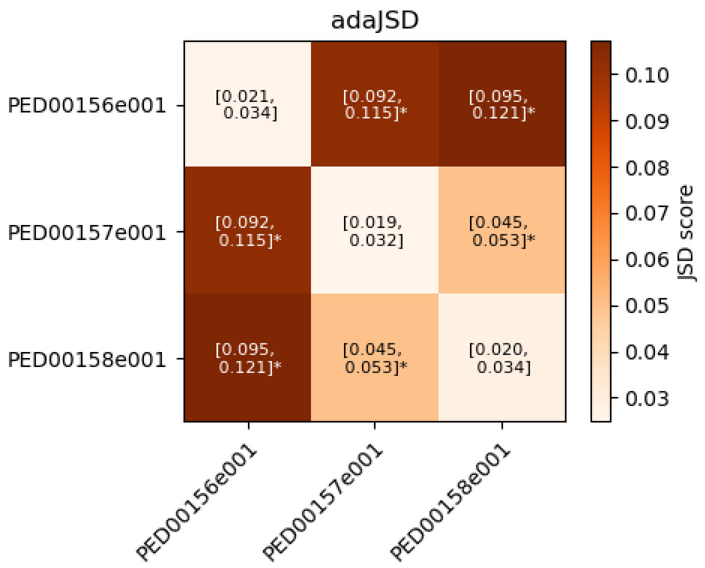

Supplementary Figure 2. It shows the result of bootstrapping by providing the confidence intervals and the statistical significance for both intra-ensemble and inter-ensemble comparisons based on adaJSD score. In this example, while PED00157 and PED00158 have low adaJSD scores, these scores appear to be significantly higher than those obtained by auto-comparisons, hinting at a systematic difference.

Visualization

In IDPET, a dedicated module has been developed specifically for data visualization, offering multiple modes to analyze conformational ensembles effectively. Each visualization method includes customizable options, allowing users to easily adjust and refine the plots according to their needs. The table below summarizes the different visualization modes available in IDPET:

Type of analysis	Type(s) of visualization	Main options supported
Distributions of ensemble properties<br>- *Rg*<br>- *Asphericity*<br>- *Prolateness*<br>- *End-to-end distance*<br>- *Global SASA*	- Histogram<br>- Violin plot	- number of bins<br>- mean/median indicator<br>- multiple plots<br>- color
Ensemble comparison	- comparison matrix heatmap	
Dimensionality reduction	- Density plot<br>- Scatter plots	- color by different labels
DSSP	- relative DSSP content plot based on selected DSSP code	- DSSP code among “H” for helix, “E” for strand and “C” for coil
Distance/contact maps	- average distance map through ensembles<br>- contact probability map based on a selected contact threshold	- contact threshold<br>- turn to log-scale
Angles	- Ramachandran plot<br>- Alpha angle	
Site-specific order/flexibility parameters	- Site-specific order/flexibility parameter for each residue in the ensemble	- residue pointer
Per residue average SASA	- plot the average SASA for each residue among all conformations in the ensemble	- residue pointer

Operation	*Aβ40, n=300*	*Aβ40, n=2998*	*α-syn, n=300*	*α-syn, n=2998*
Initial download+process from PED. *Download PDB files from PED and process them with MDTraj. Performed only the first time a user retrieves the ensembles from PED.*	*-*	*105.9*	*-*	*388.3*
Ensemble loading. *Loading trajectory files, performed every time users analyze the ensembles.*	*-*	*1.0*	*-*	*3.4*
Quick global descriptors. *Run sequentially these methods of the Visualization class: radius_of_gyration, end_to_end_distances, asphericity, prolateness*	*0.5*	*1.3*	*0.7*	*2.9*
Global SASA. *Run the visualization.global_sasa method*	*15.4*	*153.3*	*56.4*	*562.6*
adaJSD. *Run visualization.comparison_matrix(score="adaJSD", bootstrap_iters=None)*	*0.1*	*0.3*	*1.3*	*4.6*
adaJSD bootstrap x 25. *Run visualization.comparison_matrix(score="adaJSD", bootstrap_iters=25)*	*4.9*	*13.7*	*64.7*	*223.9*
PCA with Ca-dist. *Run analysis.extract_features(featurization='ca_dist') and analysis.reduce_features(method='pca')*	*0.02*	*0.1*	*0.2*	*1.2*
kPCA with phi/psi. *Run analysis.extract_features(featurization='phi_psi')and analysis.reduce_features(method='kpca', circular=True)*	*0.07*	*53.4*	*0.1*	*54.2*
t-SNE selection with Ca-dist. *Run analysis.extract_features(featurization='ca_dist')and analysis.reduce_features(method='kpca', **tsne_params)*	*25.0*	*387.6*	*30.7*	*445.9*
t-SNE selection with phi/psi. *Run analysis.extract_features(featurization='phi_psi')and analysis.reduce_features(method='kpca', circular=True, **tsne_params)*	*38.4*	*1427.8*	*46.6*	*1931.3*

Supplementary Table 1. Wall-clock times for common IDPET operations. The analyses were performed using 3 PED ensembles for Aβ40 (PED00531-PED00533, with 40 residues) or α-synuclein (PED00543-PED00545, with 141 residues) with all the conformers available (2998 per ensemble) or with smaller subsamples (300 per ensemble). For all t-SNE analyses: perplexity_vals=[10, 20, 50, 100], range_n_clusters=range(2, 5, 1). All tests were run with Python 3.10 on a MacBook Air (2020) platform with a M1 processor and 16 GB RAM. Wall-clock times are reported in seconds.

Feature	IDPET	SOURSOP
Input	Supports multi-model PDB files and trajectory files (.dcd, .xtc).	Supports trajectory files (.dcd, .xtc).
Analysis pipeline	Can handle multiple ensembles, each containing a single chain.	Can handle a single ensemble containing multiple chains.
Ensemble features	Calculates common global and local structural descriptors such as radius of gyration (Rg), end-to-end distance (Ree), scaling exponent, and backbone dihedral angles.	Computes a broader range of structural features,
Visualization	Includes a built-in visualization module for plotting and interactive analysis.	Does not include a dedicated visualization module, but example Jupyter notebooks are provided
Experimental observables	Does not directly compute experimental observables.	Can provide experimental observables such as chemical shifts and PRE values.
Dimensionality reduction	Supports dimensionality reduction methods such as t-SNE and PCA..	Currently does not include dimensionality reduction features.
Ensemble comparison and validation	Provides quantitative ensemble comparison using Jensen–Shannon divergence (JSD) metrics.	Includes functions for assessing sampling quality.

Supplementary Table 2. Comparison between the IDPET and SOURSOP packages for analyzing conformational ensembles of IDPs. Various aspects of the two packages are compared. For a quantitative comparison, a Jupyter notebook demonstrating their functionalities is provided in the GitHub repository of the package.

Supplementary References:

1. Appadurai, R., Koneru, J. K., Bonomi, M., Robustelli, P. & Srivastava, A. Clustering Heterogeneous Conformational Ensembles of Intrinsically Disordered Proteins with t-Distributed Stochastic Neighbor Embedding. *J. Chem. Theory Comput.* **19**, 4711–4727 (2023).
2. Lindorff-Larsen, K. & Ferkinghoff-Borg, J. Similarity measures for protein ensembles. *PloS One* **4**, e4203 (2009).
